# Adipose tissue-derived mesenchymal stem cells and platelet-rich plasma: stem cell transplantation methods that enhance stemness

**DOI:** 10.1186/s13287-015-0217-8

**Published:** 2015-11-05

**Authors:** Morikuni Tobita, Satoshi Tajima, Hiroshi Mizuno

**Affiliations:** Department of Plastic and Reconstructive Surgery, Juntendo University School of Medicine, 2-1-1 Hongo, Bunkyo, Tokyo 1138421 Japan

## Abstract

Because of their ease of isolation and relative abundance, adipose-derived mesenchymal stem cells (ASCs) are a particularly attractive autologous cell source for various therapeutic purposes. ASCs retain a high proliferation capacity in vitro and have the ability to undergo extensive differentiation into multiple cell lineages. Moreover, ASCs secrete a wide range of growth factors that can stimulate tissue regeneration. Therefore, the clinical use of ASCs is feasible. However, the potential of ASCs differs depending on the donor’s medical condition, including diseases such as diabetes. Recent studies demonstrated that ASCs from diabetic donors exhibit reduced proliferative potential and a smaller proportion of stem cell marker-positive cells. Therefore, to ensure the success of regenerative medicine, tissue engineering methods must be improved by the incorporation of factors that increase the proliferation and differentiation of stem/progenitor cells when autologous cells are used. Platelet-rich plasma (PRP), which contains high levels of diverse growth factors that can stimulate stem cell proliferation and cell differentiation in the context of tissue regeneration, has recently been identified as a biological material that could be applied to tissue regeneration. Thus, co-transplantation of ASCs and PRP represents a promising novel approach for cell therapy in regenerative medicine. In this review, we describe the potential benefits of adding PRP to ASCs and preclinical and clinical studies of this approach in various medical fields. We also discuss the mechanisms of PRP action and future cell-based therapies using co-transplantation of ASCs and PRP.

## Review

Mesenchymal stem cells (MSCs) represent independent populations of stem cells with self-renewing properties and an established multipotent differentiation profile in vitro [1, 2]. Furthermore, they have several advantages in regard to clinical applications for the purpose of repairing or regenerating damaged tissues, especially because they avoid the ethical issues raised by the use of embryonic stem cells [3].

Numerous clinical studies using MSCs have been performed in various fields. Autologous MSCs represent an attractive source for cell-based regenerative medicine because these immature cells are present in the bone marrow, peripheral blood, menstrual blood, and nearly all adult tissues (for example, adipose tissue, synovium, dermis, periosteum, and deciduous teeth), as well as in solid organs (for example, liver, spleen, and lung) [4–6]. In particular, adipose-derived stem cells (ASCs) obtained from lipoaspirates have multilineage potential; that is, they are capable of differentiating into adipogenic, chondrogenic, myogenic, osteogenic, and neurogenic cells [7, 8]. Thus, ASCs could be used in clinical applications for the repair of damaged tissues, as well as for angiogenic therapy. Injection of human ASCs was recently shown to induce osteoid matrix formation and improve neovascularization in an ischemic hind limb in immunotolerant mice [9–11]. Similarly, ASCs can increase the functional capacity of damaged skeletal muscle in vivo [12]. Moreover, ASCs are abundant and easy to harvest from patients’ inguinal fat pads.

However, although cell-based therapies using ASCs are a promising approach for regenerating damaged tissues, the detailed mechanisms underlying the regenerative pathways of transplanted ASCs are not clearly understood. Recent publications have suggested that ASC differentiation may not be the main regenerative mechanism in cell therapy, although the multipotent characteristics of these preparations have been demonstrated in vitro and have attracted the greatest attention from the standpoint of their use in tissue engineering approaches. Most of the beneficial effects of stem cells might be attributed to soluble factors released from stem cells [3]. However, several groups report that ASCs derived from different tissues not only share many similarities but also seem to exhibit differences in terms of marker expression and biological properties [3]. Furthermore, the biological properties of ASCs are influenced by systemic disease such as diabetes. ASCs isolated from type 2 diabetics exhibit elevated levels of cellular senescence and apoptosis, as well as altered differentiation capacity [13]. Similarly, Cianfarani et al. [14] reported that stromal vascular fractions (SVFs) isolated from diabetic animals exhibit several alterations. In material obtained from diabetic donors, the percentage of cells expressing stem cell-specific membrane markers in SVFs and cultured cells is reduced. Moreover, the levels of vascular endothelial growth factor (VEGF)-A, hepatocyte growth factor (HGF), and insulin-like growth factor (IGF)-1 in the conditioned medium of diabetic ASCs are also reduced. These observations suggest that diabetic ASCs suffer from impairments in the ability to produce or release factors that mediate cell signaling [15].

Recently, platelet-rich plasma (PRP) was introduced in tissue engineering as a source of large quantities of growth factors, and this material has been applied as a novel matrix to enhance the properties of transplanted cells. PRP has been used clinically in humans since the 1970s for its wound-healing properties, which are attributed to its high levels of growth factors and secretory proteins [16]. The growth factors in PRP promote the recruitment, proliferation, and differentiation of cells involved in tissue regeneration [17].

Preclinical studies using ASCs and PRP in combination have been conducted in the contexts of periodontal tissue engineering [18, 19], wound healing [20], tendon repair [21], and bone regeneration [22]. These reports demonstrate the potential of PRP as a cell carrier (scaffold) to increase the potential of the transplanted cells used in stem cell therapies. Therefore, it is possible that PRP could contribute to stem cell therapies.

The purpose of this article is to describe the basic science of ASCs and PRP, the potential benefits of adding PRP to ASCs, and preclinical and clinical studies in various medical fields. We also discuss the mechanisms of PRP action and future cell-based therapies using co-transplantation of ASCs and PRP.

### Characterization of adipose-derived stem cells

Adipose tissue contains SVFs including pre-adipocytes, fibroblasts, vascular smooth muscle cells, endothelial cells, resident monocytes/macrophages, lymphocytes and ASCs, and is composed mainly of fat cells organized into lobules [23, 24].

Stem cell yields from adipose tissue are greater than those from other stem cell reservoirs, making them especially suitable for use in regenerative medicine. Routinely, 10^7^ adipose stromal/stem cells can be isolated from 300 ml of lipoaspirate with greater than 95 % purity [23, 25]. ASCs comprise approximately 2 % of the nucleated cells in processed lipoaspirate, and the yield of ASCs is approximately 5000 fibroblast colony-forming units (CFU-F) per gram of adipose tissue, compared with approximately 100 to 1000 CFU-F per ml of bone marrow [26].

In 2006, the International Society for Cellular Therapy proposed minimal phenotypic criteria for the definition of cultured MSCs. The main criteria for MSCs are: (1) adhesion to plastic; (2) more than 95 % of the MSC population must express CD73, CD90, and CD105 and their population must lack expression of CD34, CD45, CD11b or CD14, CD79 or CD19, and HLA class II (less than 2 % should express these); and (3) tri-lineage differentiation potential [27, 28]. In its position statement, the Society also specified CD34 as a negative marker for MSCs [27], but recent reports show that this marker must be evaluated in the context of the tissue from which the MSCs were isolated.

Reports of the percentage of SVF cells expressing CD34 vary greatly [29–32]. Up to 85 % of the cells in SVFs express CD34 [30, 33, 34]. Two days after plastic adherence, more than 95 % of cells express CD34, co-express mesenchymal (CD10/CD13/CD90) and pericytic markers (CD140a and -b), and are CD31–/CD45– [35]. Furthermore, distinct CD34+ subpopulations have been described [30, 31, 36].

### Factors that decrease stemness in adipose-derived stem cells

ASCs can be expanded ex vivo in a relatively short period of time [37–39]; however, their ‘stemness’, defined by their potential to proliferate and differentiate, gradually decreases during serial passage [37].

The differentiation capacity of ASCs may not be involved in their primary regenerative mechanism in cell therapy; however, the multipotent character of these preparations has been demonstrated in vitro and is the main focus of attention in the context of their use in tissue engineering [3]. ASCs secrete cytokines, growth factors, and bioactive molecules with trophic paracrine effects in response to local microenvironmental cues, and these factors are likely to mediate the main mechanisms underlying the regenerative and repair potential of these cells [40]. However, a large number of studies show that cultured ASC preparations are heterogeneous and consist of different populations of stem and progenitor cells with self-renewal properties and multipotent differentiation profiles [2]. The heterogeneity of ASC preparations may be due to various causes, including inter-donor differences in age, body mass index, gender, ethnicity, and disease status [1]. Sethe et al. [41] reported that MSCs from older donors show no spindle-shaped morphology in culture compared with MSCs from younger donors. In another report, Xu et al. [42] demonstrated that the osteogenic differentiation potential of ASCs is related to donor age. Body mass index correlates negatively with the number of stromal cells per gram and their differentiation capacity [1]. In addition, Gimble et al. [43] suggested that brown adipose tissue and white adipose tissue show different capacities with regard to cell proliferation and yield of stem cells. Furthermore, epigenetic changes affect stem cell growth and cell differentiation potential. Yan et al. [44] reported that pretreatment with 5-azacytidine improved proliferation and osteogenic differentiation of ASCs from older donors.

In particular, systemic diseases such as diabetes influence the properties of ASCs. Because the hyperglycemic diabetic environment may impact aspects of stemness, including the phenotype, morphology, and differentiation potential of ASCs, the potential use of autologous cell therapies in diabetic patients has caused controversy. Several studies demonstrate that the ASCs from diabetics have impaired function relative to ASCs from non-diabetic donors [15]. In ASCs from diabetic rats, for example, MSC markers are downregulated, and viability and differentiation potential are reduced [45, 46]. Based on the reduced proliferative potential and migration and limited therapeutic potential of autologous ASCs when administered to wounds of diabetic mice, some authors have questioned the efficiency of autologous therapies in diabetic patients [14, 47]. In an in vivo study, ASCs from streptozotocin-induced type 1 diabetic mice exhibited reduced proliferative potential and migration, and diabetic ASCs released lower amounts of HGF, VEGF-A, and IGF-1 [14]. Although autologous ASC administration improves healing in diabetic skin repair [48], functional impairment in resident and recruited cells strongly contributes to delayed wound healing in diabetic subjects [49–54]. Therefore, it is essential to evaluate the impact of the diabetic milieu on clinical applications of ASCs. In a clinical trial in which autologous ASCs were used for the treatment of critical limb ischemia, ASCs from diabetic patients had fibrinolytic activity, which was suggested to cause peripheral microthrombosis [55].

### Factors that increase stemness in adipose-derived stem cells, and the potential of platelet-rich plasma

Recent reports suggested that certain growth factors, such as vascular VEGF, fibroblast growth factor (FGF)-2, FGF-4, FGF-6, FGF-7, FGF-9, FGF-17, transforming growth factor (TGF)-beta1, TGF-beta2, HGF, keratinocyte growth factor, platelet-derived growth factor AA, and IGF-1, regulate the maintenance of ASC stemness [37]. These factors affect a plethora of responses such as angiogenesis, cellular migration, apoptosis, proliferation, and differentiation [56–59]. In particular, the proliferation of ASCs is regulated by paracrine factors such as FGF-2, FGF-4, interleukin (IL)-6, and stromal-derived factor 1, whereas FGF-2, endothelial growth factor, TGF-beta, and other factors are involved in differentiation [60, 61].

Platelets contain critical growth factors and mediators of tissue repair pathways. Activation of platelets with calcium chloride induces immediate platelet growth factor release in vitro [62]. PRP obtained from autologous blood contains a high concentration of stored autologous growth factors. Exposure of PRP to calcium chloride induces platelet degranulation. Several studies describe the potential benefits of using PRP in tissue regeneration; in particular, PRP therapy has been proposed in wound healing. Moreover, because PRP is able to stimulate proliferation of undifferentiated stem cells as well as cell differentiation, it might be used in conjunction with stem cell transplantation to promote tissue regeneration [63–66].

However, although the biological mechanism and clinical effect of PRP remain poorly understood, some studies on the mechanism of action of PRP have been reported recently. Andia et al. [67] demonstrated that human PRP induces an immunomodulatory and proangiogenic phenotype consistent with healing mechanisms of inflamed tenocytes in vitro. The expression of some crucial inflammatory molecules, including IL-6 and IL-8, was downregulated in response to PRP treatment.

Furthermore, various methods for manipulating PRP have been reported. For example, the platelet concentration in plasma [68], the volume of PRP transplantation, and the method of activation contribute to the effect of PRP on cell proliferation and differentiation directly. For these reasons, appropriate methods when using PRP are still controversial.

**Fig. 1:**
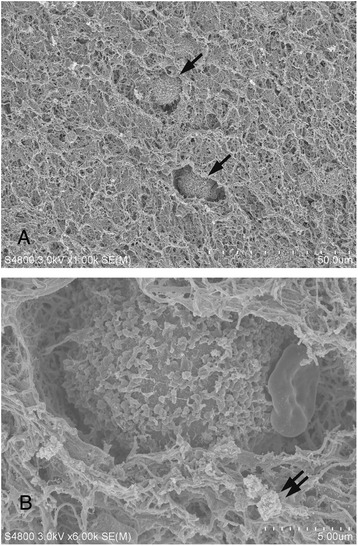
**a** Low-magnification scanning electron microscopy (SEM) image of a mixture of adipose-derived stem cells (ASCs) with activated platelet-rich plasma (PRP). Some ASCs are arranged three-dimensionally in the fibrin network of the activated PRP. **b** High-magnification SEM image of panel **a**. Some platelets reside around the ASCs. Arrows indicate ASCs and double arrows indicate platelets

On the other hand, the fibrin network of PRP has the potential to serve as a scaffold. We have shown that this network can hold cells and platelets in a three-dimensional arrangement within the PRP (Fig. 1). This cell–PRP interaction may increase stemness and prolong the survival time and rate of cells in the PRP. Therefore, the co-transplantation of ASCs and PRP represents an attractive approach for autologous cell therapies.

### Preclinical and clinical studies using adipose-derived stem cells and platelet-rich plasma

Recently, research comparing ASCs alone or in combination with PRP have been reported. Kim et al. [69] compared fat graft survival with PRP, ASCs, and SVFs in a mouse model. Transplanted fat tissue with ASCs or SVFs was effective in preventing volume reduction of fat tissue compared with conventional fat graft or adding PRP. In another study, human ASCs or adipocytes were cultured with PRP in vitro [70]. The results showed that ASC viability was strongly increased in the presence of 5 % or 20 % PRP. Furthermore, levels of IL-6, IL-8, IL-10, VEGF and interferon-γ were significantly increased in PRP-treated adipocytes [70]. Atashi et al. [71] reported that 20 % PRP was the most effective concentration to promote ASC proliferation. Recently, Xu et al. [42] reported that activated PRP promoted proliferation and differentiation of human ASCs in vitro. Interestingly, the effects of PRP on ASC proliferation and osteogenic differentiation were dose-dependent.

Recently, co-transplantation of ASCs and PRP has been extensively investigated, and the role of PRP factors as powerful paracrine effectors in ASC transplantation has been demonstrated in preclinical and clinical studies (Table 1).

**Table 1:** Preclinical and clinical studies using adipose-derived stem cells and platelet-rich plasma

Type of cells (SVFs or ASCs)	Species of cells	How to use the PRP	Animal model/disease	Results	Reference
Preclinical studies					
ASCs	Human	Co-administration	Mouse full thickness wound model	Co-administration of PRP and ASCs in the wound beds increased ASC survival and enhanced arteriole formation in wounds	[20]
ASCs	Rat (Wistar)	Co-administration	Osteonecrosis of the jaw	The combination of ASCs and PRP prevented frequency of bisphosphonate-related osteonecrosis of the jaw	[73]
ASCs	Rabbit	Co-transplantation	Achilles tendon injured model	ASCs differentiated into tenocytes	[21]
ASCs	Human	ASCs were treated with 15 % PRP in culture flask	Mouse model of articular cartilage injury	PRP-treated ASCs improved healing of injured articular cartilage	[72]
ASCs	Rat (Fischer)	Co-transplantation	Rat calvarial defect	Regenerated volume of bone was significantly greater than in the PRP-treated group and ASCs/collagen gel-treated group 8 weeks after transplantation	[22]
ASCs	Rat (Wistar)	Co-transplantation	Rat periodontal tissue defect	Alveolar bone, periodontal ligament-like structures, and cementum-like structures were observed in the periodontal tissue defect 8 weeks after transplantation	[18]
ASCs	Canine	Co-transplantation	Canine periodontal tissue defect	Periodontal tissue regeneration was observed in the bifurcation defect 8 weeks after transplantation	[19]
SVFs	Human	Co-transplantation	Rat periodontal tissue defect	Human SVFs have the potential to regenerate periodontal tissue	[24]
Clinical studies					
ASCs	Human	Co-transplantation	Osteoarthritis	18 patients were treated. Intra-articular injection of a combination of ASCs and PRP effectively reduced pain and improved knee function in patients being treated for knee osteoarthritis	[74]
SVFs	Human	Co-transplantation	Articular joints	91 patients were treated with autologous SVFs with PRP over 2 years	[75]

*ASC* adipose-derived stem cell; *PRP* platelet-rich plasma; *SVF* stromal vascular fraction

Numerous preclinical studies have demonstrated the efficacy of co-transplantation of ASCs and PRP in a wide range of model systems. Van Pham et al. [72] transplanted ASCs cultured with 15 % PRP into the articular cartilage injury model of NOD/SCID mice. Their results showed that PRP-pretreated ASCs improved healing of injured articular cartilage more effectively than untreated ASCs. In a bone regeneration study, Tajima et al. [22] transplanted rat ASCs and PRP into a rat calvarial defect model. They found that co-transplantation of ASCs and PRP significantly improved bone regeneration; furthermore, 8 weeks after transplantation, the volume of regenerated bone was significantly greater when PRP and ASCs were transplanted together than when PRP or ASCs in collagen gel were used alone. In another study, Tobita et al. [18] transplanted a combination of rat ASCs and PRP into a periodontal tissue defect in Wistar rats. Eight weeks after this treatment, histological observation revealed regeneration of alveolar bone, periodontal ligament-like structures, and cementum-like structures in the periodontal tissue defect. Likewise, when canine-derived ASCs were transplanted with PRP, regeneration of these periodontal tissues was greatly improved relative to PRP-treated or saline-treated control subjects 8 weeks after transplantation [19]. Tobita and Mizuno [24] transplanted uncultured human SVFs, taken from subcutaneous fat tissue, along with PRP into a periodontal tissue defect in nude rats; this treatment resulted in extensive improvement of periodontal tissue 8 weeks after transplantation. A recent study reported the transplantation of a combination of ASCs and PRP for treatment of bisphosphonate-related osteonecrosis of the jaw in a rat model [73]. The results demonstrated that a lower frequency of osteonecrosis was associated with the combination of ASCs and PRP.

Likewise, in clinical studies, combined transplantation of ASCs and PRP has shown great promise. Koh et al. [74] injected a combination of ASCs and PRP into 18 patients with osteoarthritis or degenerative cartilage, and found that this treatment effectively reduced pain and improved knee function in patients being treated for knee osteoarthritis. Pak et al. [75] investigated the safety of implanting autologous SVFs and PRP into articular joints; in this study, 91 patients were treated with a combination of autologous SVFs with PRP over the course of 2 years.

## Conclusion

ASCs hold great potential for use in stem cell therapy. After being transplanted, however, ASCs face a complex and hostile environment in which local hypoxia, oxidative stress, and inflammation may lead to cell loss or death on a large scale. Furthermore, the stemness properties of ASCs are influenced by the disease state of the donor. Insufficient retention and survival of transplanted ASCs can dramatically reduce their therapeutic effects [76]. Therefore, tissue engineering approaches need to be dramatically improved by the addition of adjuncts that increase the proliferation and differentiation of ASCs. In this regard, PRP is an attractive cell-maintained biomaterial, and the activated PRP scaffold can enhance the stemness properties of ASCs, although further analysis and investigation are needed to establish novel cell therapies.

In particular, risk assessments of cell transplantation in clinical studies are especially important because clinical efficacy and safety depend on the manipulation of various factors, culture conditions, and quality risk management.

The evidence compiled to date suggests that this combination treatment represents a promising approach in various fields of medicine and dentistry.

## Note

This article is part of a thematic series ‘Mesenchymal Stem/Stromal Cells—An update’. Other articles in this series can be found at http://www.biomedcentral.com/series/mesenchymal

## Abbreviations

ASC: Adipose-derived stem cell
CFU-F: Fibroblast colony-forming units
FGF: Fibroblast growth factor
HGF: Hepatocyte growth factor
IGF: Insulin-like growth factor
IL: Interleukin
MSC: Mesenchymal stem cell
PRP: Platelet-rich plasma
SVF: Stromal vascular fraction
TGF: Transforming growth factor
VEGF: Vascular endothelial growth factor
